# Co-circulation of Aedes flavivirus, Culex flavivirus, and Quang Binh virus in Shanghai, China

**DOI:** 10.1186/s40249-018-0457-9

**Published:** 2018-07-16

**Authors:** Yuan Fang, Yi Zhang, Zheng-Bin Zhou, Wen-Qi Shi, Shang Xia, Yuan-Yuan Li, Jia-Tong Wu, Qin Liu, Guang-Yi Lin

**Affiliations:** 1 National Institute of Parasitic Diseases, Chinese Center for Disease Control and Prevention; WHO Collaborating Centre for Tropical Diseases; National Center for International Research on Tropical Diseases, Ministry of Science and Technology; Key Laboratory of Parasite and Vector Biology, Ministry of Health, 207 Rui Jin Er Road, Shanghai, 200025 People’s Republic of China; 2 Shanghai Medical College, Fudan University, Shanghai, 200032 People’s Republic of China

**Keywords:** Aedes flavivirus, *Aedes albopictus*, Culex flavivirus, *Culex pipiens*, *Culex tritaeniorhynchus*, Quang Binh virus

## Abstract

**Background:**

With increases in global travel and trade, the spread of arboviruses is undoubtedly alarming. Pathogen detection in field-caught mosquitoes can provide the earliest possible warning of transmission. Insect-specific flavivirus (ISFV) has been first detected in 1991 and documented worldwide in the latest ten years. Although infection with ISFVs is apparently limited to insects, an increase in the infection rate of mosquito-borne flaviviruses may be able to induce cytopathic effects in vertebrate cells during co-infection with other human pathogens. However, little is known whether ISFVs persist in most regions of China.

**Methods:**

During the mosquito activity season in 2016, a surveillance program was carried out to detect ISFVs in mosquitoes in metropolitan Shanghai, China. The presence of ISFVs was randomly tested in different species of mosquitoes using RT-PCR-based and hemi-nested PCR assays, following by the sequencing of PCR products. Sequences from positive pooled samples were compared with those deposited in GenBank. Thereafter, sequences of representative insect flaviviruses were used for further phylogenetic and molecular evolutionary analyses.

**Results:**

Our investigations showed: (1) the presence of Aedes flavivirus (AEFV) in 11/161 pooled samples (nine pools in Songjiang District, one pool in Huangpu District, and one pool in Qingpu District) of *Aedes albopictus*, (2) the presence of Quang Binh virus (QBV) in 10/195 pooled samples (all in Chongming District) of *Culex tritaeniorhynchus*; and (3) the presence of Culex flavivirus (CxFV) in 9/228 pooled samples (six pools in Pudong New Area, two pools in Huangpu District, and one pool in Chongming District) of *Cx. pipiens*. Furthermore, phylogenetic analyses of the gene sequences of envelope proteins indicated that Shanghai CxFV strains belonged to the Asia/USA genotype. The overall maximum likelihood estimation values (and 95% confidence interval) for CxFV, QBV, and AEFV in mosquitoes collected in Shanghai in 2016 were 1.34 (0.66–2.45), 1.65 (0.87–2.85), and 1.51 (0.77–2.70) per 1000, respectively.

**Conclusions:**

This study reveals the presence and the geographical distribution of ISFVs, and determines the genetic variation and the infection rate of ISFVs in Shanghai, China. At least, three insect flaviviruses including ISFVs, AEFV, CxFV, and QBV, co-circulate in this area. To our knowledge, this is the first report of AEFV in China.

**Electronic supplementary material:**

The online version of this article (10.1186/s40249-018-0457-9) contains supplementary material, which is available to authorized users.

## Multilingual abstracts

Please see Additional file 1 for translations of the abstract into the five official working languages of the United Nations.

## Background

Transmitted by arthropods, arboviruses have become a significant cause of public health issues worldwide, with the potential of leading to unprecedented spread and causing epidemics. Most arboviruses of medical significance belong to three genera: *Flavivirus* (family Flaviviridae), *Alphavirus* (family Togaviridae), and *Orthobunyavirus* (family Peribunyaviridae) [1]. The genus *Flavivirus* contains close to 80 enveloped, single-stranded, positive-sense RNA viral species that exhibit a wide range of geographic distributions and diverse relationships with their hosts. Most known flaviviruses are associated with human diseases, and are transmitted to vertebrate hosts by mosquitoes or ticks. Their genomes, which vary in length from 9 to 13 kb, are made up of ten functional genes, of which three encode structural proteins (capsid, C; pre-membrane, prM; and envelop, E), and the remaining seven encode non-structural proteins (NS1, NS2a, NS2b, NS3, NS4a, NS4b, and NS5) [2]. According to known host types and phylogenetic relationships, members of the genus *Flavivirus* are divided into four groups. These include: 1) mosquito-borne flaviviruses—such as Dengue virus (DENV), Japanese encephalitis virus (JEV), Yellow fever virus, West Nile virus (WNV), and Zika virus—the majority of which are zoonoses that infect a range of vertebrate hosts via mosquitoes and can cause public health issues; 2) tick-borne flaviviruses, like the tick-borne encephalitis virus; 3) no-known vector vertebrate flaviviruses, which can replicate in vertebrate cells but with no known arthropod hosts; and 4) insect-specific flaviviruses (ISFVs), which are specific for mosquitoes and unable to replicate in mammalian cells [2–4]. The ISFVs are speculated to represent the genetic primordial form of the genus [5].

Based on the molecular structures and evolution rates of known flaviviruses, approximately 2000 unidentified flaviviruses are estimated to exist [6]. Cell fusing agent virus (CFAV), originally discovered and characterised by syncytium formation of cytopathic effect (CPE) in the *Aedes aegypti* cell line [7], is recognised as the first member of the ISFV group, and was isolated from several species of natural mosquitoes from Puerto Rico in 2006, including *Ae. aegypti*, *Ae. albopictus,* and *Culex* sp. [8]. The second member of this group, Kamiti River virus (KRV), isolated from *Ae. macintoshi* in Central Province, Kenyan in 2003, is considered to be the first ISFV isolated from nature [9]. Culex flavivirus (CxFV) was first isolated from *Cx. pipiens* in 2007 in Japan and Indonesia [5]. Moreover, CxFVs have subsequently been detected in *Cx. pipiens*, *Cx. quinquefasciatus*, *Cx. restuans*, *Cx. tritaeniorhynchus*, and *Anopheles sinensis* in Japan, the USA, China, Uganda, Mexico, and Brazil [1, 10–16]. Several novel flaviviruses have been isolated and characterised as ISFVs more recently. They include: Aedes flavivirus (AEFV), which was detected in *Ae. flavopictus* and *Ae. albopictus* mosquitoes from Japan [3]; Quang Binh virus (QBV), isolated from *Cx. tritaeniorhynchus* from Vietnam [17]; Nakiwogo virus (NAKV), isolated from *Mansonia africana nigerrima* from Uganda [12]; Calbertado virus, detected primarily in *Cx. trasalis* in Western Canada [18]; Spanish Culex flavivirus, detected in *Cx. theileri* and *Cx. pipiens* from Spain [19]; Ochlerotatua flavivirus (OcFV) detected in *Ochlerotatua caspius* from Italy, Portugal and Spain [19–21]; Hanko virus (HANKV), isolated from *Oc. punctor* and/or *Oc. caspius* from Finland [22]; and Yamadai flavivirus (YDFV), isolated from *Cx. tritaeniorhynchus* from Japan [23]. Additionally, flavivirus RNAs have been detected in sandflies [24], and one was also detected in chironomids, another family from the order Diptera [25].

Culex flavivirus was the first ISFV reported in China, where it was isolated in 2012 from *Cx. pipiens* in Dongming, Shandong Province [13]. Since then, an increasing number of CxFV detections have been documented in China [14, 16, 26]. Other ISFVs that have been isolated and characterised in China are the Quang Binh-like virus [27] and Yunnan Culex flavivirus (YNCxFV) [26]. Both of their nucleotide sequences were 83% identical to that of QBV, a species identity percentage that is just below the cut-off value of 84% proposed by Kuno [28], and were detected mainly in *Cx. tritaeniorhynchus*, the same host in which QBV has been detected [17, 26, 27]. However, it is unclear whether they are novel species of ISFVs, because, they were collected from the Yunnan Province, which borders Vietnam, the country where QBV was discovered [26]. In order to resolve this issue, neutralisation tests are recommended, when nucleotide sequence identity is higher than 80% [28].

The ISFV CPE can be weak, strain-dependent, or only visible after a number of blind passages [11, 29], indicating that a fine balance exists between virus escape mechanisms and the mosquito immune system. In contrast to most flaviviruses, ISFVs show peculiar characteristics. The sequences of some ISFVs can integrate into the genomes of mosquitoes both in the field and in laboratory cell lines, mainly occurring in *Aedes* mosquitoes [19, 30, 31]. Thus, when attempting to detect ISFVs, nucleic acid extracts should be treated with DNase to exclude interference by integrated DNA forms of the virus.

Insect-specific flaviviruses have been propagated experimentally only in mosquito cells, and are probably transmitted vertically because eggs, larvae, female, and male mosquitoes can be infected in nature [1, 8, 9, 11, 32, 33] and may be transmitted horizontally through the feeding process in the field [29]. It is apparent that ISFVs are unable to affect the health of birds, domestic animals, and human beings. However, they are carried by invasive mosquitoes and likely do not cross-react with known pathogenic flaviviruses [1]. Recent studies, both in the field and in the laboratory, have shown that co-infection with CxFV increases WNV infection rate [34, 35]. Alternatively, the possibility that CxFV can be attributed to a decrease in WNV morbidity in the Yucatan Peninsula of Mexico, through reducing the number of available competent vectors has been speculated [1]. Whether superinfection exclusion of pathogenic arbovirus occurs in invasive mosquitoes infected with ISFV requires further investigation. Immunological research has shown that, during co-infection by both human pathogens and ISFVs, ISFVs may induce vertebrate cell CPE when innate immunity fails due to the activity of human pathogens [36]. In contrast, it has also been proposed that ISFVs may modulate, or even suppress the immune responses of mosquitoes, thereby making the mosquitoes more susceptible to infection by a broad range of human pathogens [35].

Clearly, research on ISFVs is progressing rapidly and expanding on a worldwide scale. ISFVs in China are far more diverse than is currently reported. However, in most parts of China, ISFVs have not been studied. The purpose of this study is to identify the presence and the infection rate of ISFVs in field-caught mosquitoes in the municipality of Shanghai, China.

## Methods

### Survey area and mosquito collection

A surveillance program for vector pathogen, involving the collection of mosquitoes and detection of ISFVs studied the distribution and diversity of ISFVs in Shanghai from June to October 2016. Located in the Yangtze River Delta, Shanghai sits on the south edge of the estuary of the Yangtze in the middle portion of the East China coast. The region has a temperate climate, which is suitable for the reproduction of mosquitoes. Shanghai is at risk for transmission of mosquito-borne diseases, especially since it is the centre for economic trade and tourism, and has an abundance of migratory birds. The dominant mosquito species detected in this area are *Ae. albopictus*, *Cx. pipiens*, *Cx. tritaeniorhynchus*, and *An. sinensis* [37]. In this survey, six different districts, Huangpu District, Songjiang District, Jiading District, Qingpu District, Chongming Districtrict, and Pudong New Area, were chosen for the field research, covering different types of ecological environments, including urban areas, suburban areas, rural areas, and even conservation areas. During the surveillance period, two sampling methods were used to collect mosquitoes. CO_2_-baited traps were hung from sunset to sunrise in five monitoring sites of each district. Direct aspiration by humans was also used to bait mosquitoes after sunset for fifteen minutes in ten monitoring sites of each district. These two methods were carried out three times at even distributed days throughout the month.

### ISFV identification

After collection, mosquitoes were identified using morphological characteristics according to the national key [38]. Some morphological confused specimens were determined by molecular methods, as reported previously [39, 40]. Mosquitoes were then pooled by species, sex, and date and location of collection, with one to 50 individuals per pool. Pooled mosquitoes were stored in 2 ml sterile plastic tubes containing 75% alcohol and frozen at − 20 °C. Sampled pools of mosquitoes were homogenised in a frozen block using a Mixer Mill (Jingxin, Shanghai, China) with one 3 mm and one 5 mm stainless-steel bead added. If fewer than 20 mosquitoes were present in the sample, 450 μl of TRIzol (Invitrogen, Carlsbad, CA) was added, and if more than 20 mosquitoes were present, 600 μl of TRIzol was added to the sample. The samples were then centrifuged at full speed (12 700 rpm) for 10 mins at 4 °C. Then, 350 μl of supernatant from each ground sample was added to the processing cartridge and placed into to the MagNA Pure 96 System (Roche, Basel, Switzerland) for automated RNA extraction. The MagNA Pure 96 Cellular RNA Large Volume Kit, including DNase to obtain DNA-free RNA was used for the RNA extraction according to the manufacturer’s instructions, with a final elution volume of 50 μl. First-strand cDNA was synthesised by RT-PCR using Takara PrimeScript RT reagent Kit with gDNA Eraser (TAKARA BIO, Shiga, Japan). After the RT reactions, PCR amplification targeting mosquito 18S rRNA was implemented with primers 18S417 and 18S920c [41] to verify the integrity of the RNA in each pool. If the control amplification was successful, the cDNA was amplified by hemi-nested PCR using a set of primers (cFD2 and MAMD, cFD2 and FS778) for the detection of the partial NS5 gene of flaviviruses as reported [42]. With the purpose to identify the genotypes of the CxFVs in the samples, we used CxFV-E forward and reverse primers (CxFV-E-F: 5′-ACTGGTGACGTTCAAGGCCATAAG-3′; CxFV-E-R: 5′-GCCGTGATCAGGTGCTGGTCATCG-3′), which target the E gene [43]. Amplified products were separated by agarose gel electrophoresis, then purified and sequenced in both directions by Sangon Biotech (Shanghai, China). Sequences were compared with those available in GenBank database using the BLAST program.

### Phylogenetic analysis

Multiple sequence alignments were generated with the relevant ISFV genomes obtained from GenBank and this study using ClustalW2 [44] with default settings, and were manually adjusted if necessary. Neighbour-joining trees were established following the Kimura’s two-parameter (K2P) distance model [45] with 1000 bootstrap replications using MEGA v7.0 software [46]. Based on the Akaike Information Criterion, the best-fit model for the alignment was determined using Modeltest 3.7, in cooperation with PAUP* v4.0b10 [47]. Consequently, the construction of the maximum likelihood (ML) and Bayesian likelihood was completed under the GTR + I + G model for both the NS5 gene and CxFV E gene. The ML tree was performed by MEGA v7.0 software, with 1000 bootstraps. The Bayesian tree was built with MrBayes v3.2.1 [48], run for 10 million generations, with the first 25% generations discarded as burn-in. The trees were unrooted to provide the least biased topology, and visualised using Figtree v1.4.2 (http://tree.bio.ed.ac.uk/software/figtree/).

### Calculation of infection rates

The sizes of the pools of collected mosquitoes varied considerably. For this reason, infection rates were calculated by Bias-corrected maximum likelihood estimation (MLE) and minimum infection rate (MIR) using the Excel add-in PooledInfRate v4 statistical software package [49]. The rates are expressed as the number of infected mosquitoes per 1000 collected mosquitoes.

## Results

### Detection of ISFV RNA in mosquito pools collected in Shanghai

A total of 3249 mosquitoes of the species *Ae. albopictus*, 3370 of the species *Cx. pipiens*, and 6045 of the species *Cx. tritaeniorhynchus* were collected in Shanghai during the active season for mosquitoes between June and October 2016. Of these, 161/440 pools of *Ae. albopictus*, 228/611 pools of *Cx. pipiens*, and 195/655 pools of *Cx. tritaeniorhynchus* were randomly tested for the presence of ISFVs. In order to ensure that pools only tested positive due to the amplification of viral RNA, but not due to the integration of ISFV sequences in the genome of the mosquitoes, RNA extraction protocols employed an additional DNAse step and nucleic acid extracts were treated with DNA Eraser before RT-PCR. The integrity of RNA extracted from the mosquito pools was checked by amplification of the mosquitoes’ 18S rRNA. This amplification was successful in 573 of the 576 pools. Sampled pools were screened for ISFVs using hemi-nested PCR. A fragment comprising 261 bp of the NS5 gene was amplified from pools positive for ISFVs. All the NS5 genes of the 30 ISFVs obtained in this study were detected from female mosquito pools. These sequences have already been deposited in GenBank. Details of their collection information and GenBank accession numbers are listed in Table 1.

**Table 1:** Summary of the insect-specific flaviviruses detected from different mosquito pools, captured in Shanghai, during June to October in 2016

Strain	Virus	Host	Collection date	Geographic location	Survey site type	GenBank ID	
						NS5	E
SB5_16–6-S-B-2-JG-3	AeFV	*Aedes albopictus*	14-Jun-2016	Songjiang District	Office workspace	MG602480	
SC6_16–6-S-B-4-JM-4	AeFV	*Ae. albopictus*	16-Jun-2016	Songjiang District	Suburban residential area	MG602481	
SA1_16–6-S-B-1-JM-1	AeFV	*Ae. albopictus*	14-Jun-2016	Songjiang District	Suburban residential area	MG602482	
SC3_16–6-S-B-4-JM-1	AeFV	*Ae. albopictus*	16-Jun-2016	Songjiang District	Suburban residential area	MG602483	
SA7_16–6-S-B-1-XX-3	AeFV	*Ae. albopictus*	14-Jun-2016	Songjiang District	School	MG602484	
SB4_16–6-S-B-2-JG-2	AeFV	*Ae. albopictus*	14-Jun-2016	Songjiang District	Office workspace	MG602485	
SB3_16–6-S-B-2-JG-1	AeFV	*Ae. albopictus*	14-Jun-2016	Songjiang District	Office workspace	MG602486	
SA3_16–6-S-B-1-JM-3	AeFV	*Ae. albopictus*	14-Jun-2016	Songjiang District	Suburban residential area	MG602487	
SC7_16–6-S-B-4-GY-1	AeFV	*Ae. albopictus*	16-Jun-2016	Songjiang District	Public garden	MG602488	
HL7_9-H-B-3-JM-3	AeFV	*Ae. albopictus*	22-Sep-2016	Huangpu District	Suburban residential area	MG602503	
QH8_8-Q-B-1-JM-1	AeFV	*Ae. albopictus*	8-Aug-2016	Qingpu District	Suburban residential area	MG602504	
CG8_16–7-C-Cxp-R-6-1	CxFV	*Culex pipiens*	6-Jul-2016	Chongming District	Suburban residential area	MG602489	
PJ7_16–10-P-Cxp-C-4-5	CxFV	*Cx. pipiens*	11-Oct-2016	Pudong New Area	Livestock farm	MG602490	
PJ4_16–10-P-Cxp-C-2-15	CxFV	*Cx. pipiens*	24-Oct-2016	Pudong New Area	Suburban residential area	MG602491	MG673529
PK2_16–10-P-Cxp-C-4-2	CxFV	*Cx. pipiens*	2-Oct-2016	Pudong New Area	Livestock farm	MG602492	MG673530
HK8_16–10-H-Cxp-C-1-3	CxFV	*Cx. pipiens*	12-Oct-2016	Huangpu District	Urban residential area	MG602493	
PG7_16–8 L-P-Cxp-C-2-21	CxFV	*Cx. pipiens*	22-Aug-2016	Pudong New Area	Suburban residential area	MG602494	MG673527
PF1_16–7 M-P-Cxp-C-2-27	CxFV	*Cx. pipiens*	12-Jul-2016	Pudong New Area	Suburban residential area	MG602495	MG673526
HK7_16–10-H-Cxp-C-1-1	CxFV	*Cx. pipiens*	2-Oct-2016	Huangpu District	Urban residential area	MG602496	
PJ3_10-P-Cxp-C-2-8	CxFV	*Cx. pipiens*	12-Oct-2016	Pudong New Area	Suburban residential area	MG602497	MG673528
CB8_16–8-C-Cxt-C-3-1	QBV	*Cx. tritaeniorhynchus*	12-Aug-2016	Chongming District	Rural residential area	MG602498	
CC2_16–8-C-Cxt-C-5-5	QBV	*Cx. tritaeniorhynchus*	12-Aug-2016	Chongming District	Livestock farm	MG602499	
CC1_16–8-C-Cxt-C-5-3	QBV	*Cx. tritaeniorhynchus*	12-Aug-2016	Chongming District	Livestock farm	MG602500	
CD8_16–9-C-Cxt-R-2-1	QBV	*Cx. tritaeniorhynchus*	3-Sep-2016	Chongming District	Rural residential area	MG602501	
CI5_16–7-C-Cxt-R-4-4	QBV	*Cx. tritaeniorhynchus*	25-Jul-2016	Chongming District	Livestock farm	MG602502	
ZB3_16–7-CZ-Cxt-L-N-B3	QBV	*Cx. tritaeniorhynchus*	20-Jul-2016	Chongming District	Conservation area	MG673531	
ZG2_16–8-CZ-Cxt-L-G-G2	QBV	*Cx. tritaeniorhynchus*	26-Aug-2016	Chongming District	Conservation area	MG673532	
ZG5_16–8-CZ-Cxt-L-G-G5	QBV	*Cx. tritaeniorhynchus*	26-Aug-2016	Chongming District	Conservation area	MG673533	
ZI7_16–8-CZ-Cxt-L-R-I7	QBV	*Cx. tritaeniorhynchus*	26-Aug-2016	Chongming District	Conservation area	MG673534	
ZG4_16–8-CZ-Cxt-L-G-G4	QBV	*Cx. tritaeniorhynchus*	26-Aug-2016	Chongming District	Conservation area	MG673535	

*AEFV* Aedes flavivirus, *CxFV* Culex flavivirus, *QBV* Quang Binh virus, *NS5* Non-structural 5 gene, *E* Envelope gene

**Fig. 1:**
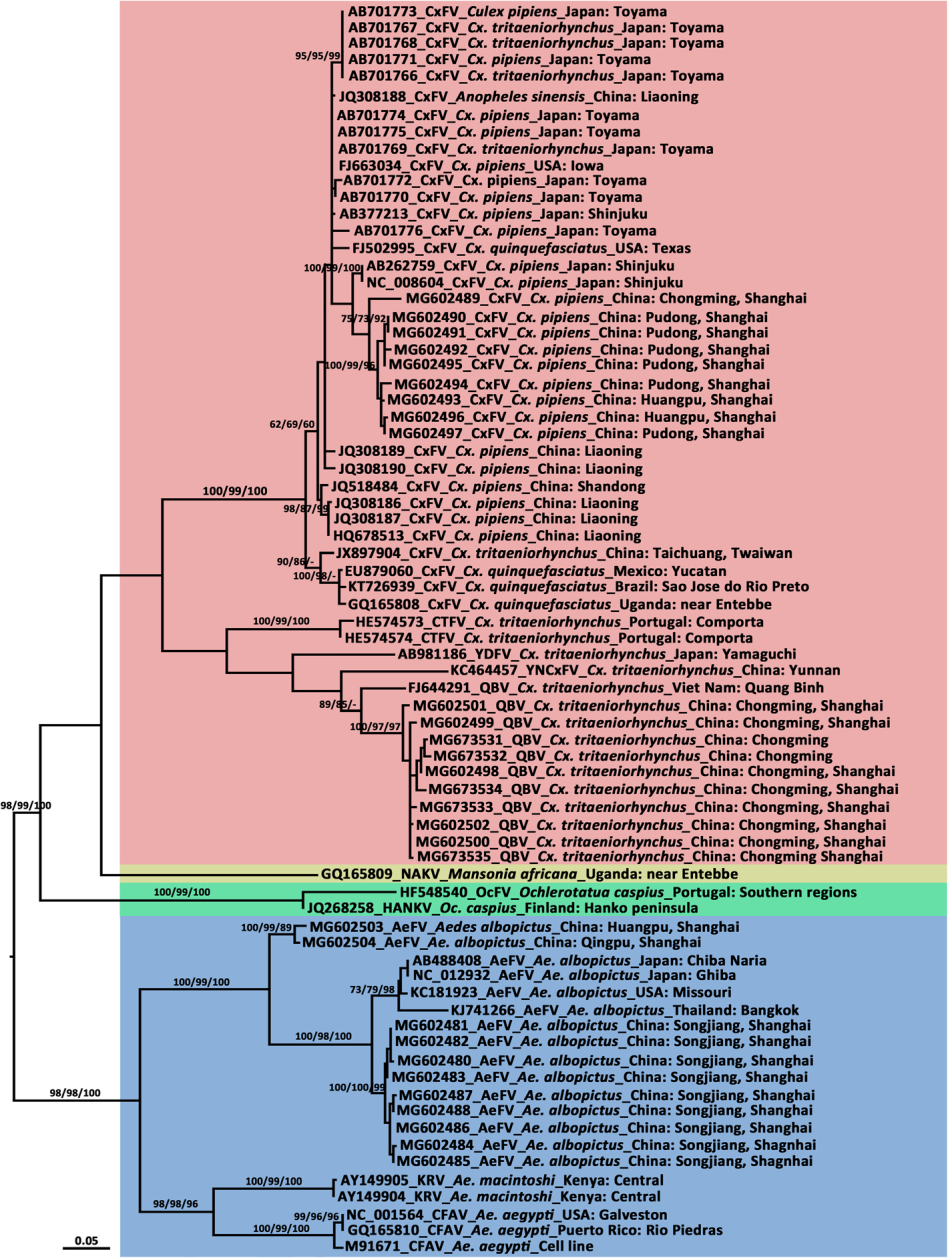
Molecular phylogeny of the 30 insect-specific flavivirus (ISFV) strains from Shanghai and other ISFVs based on partial NS5 gene (261 base pairs) sequences. The maximum likelihood tree was constructed by the GTR + I + R model. Sequences referable to the same host-genus are shown with the same colours in the tree. Bootstrap values (1000 replicates, not shown for less than 60%) of maximum likelihood, neighbour-joining, and Bayesian analyses are shown above the main lineages. The bar indicates 0.05 substitutions per site. AEFV, Aedes flavivirus; CFAV, cell fusing agent virus; CTFV, *Culex theileri* flavivirus; CxFV, Culex flavivirus; HANKV, Hanko virus; KRV, Kamiti River virus; NAKV, Nakiwogo virus; QBV, Quang Binh virus; OcFV, Ochlerotatua flavivirus; SCxFV, Spanish Culex flavivirus; YDFV, Yamadai flavivirus; YNCxFV, Yunnan Culex flavivirus. See Table 1 for information on the sequences of the Shanghai strains of ISFVs

A total of 44 nucleotide sequences from representative ISFVs that were retrieved from GenBank and the 30 sequences described here were aligned with a partial NS5 nucleotide sequence. This region was selected because the use of the NS5 gene as a target for diagnostic PCR protocols to detect flaviviruses makes partial sequences of this gene the most common. The topology of the NJ tree, ML tree, and Bayesian tree were almost identical for the major lineages, although node confidence values were slightly different among the three (Fig. 1). Hence, only the ML tree is presented with an unrooted phylogram to avoid assumptions regarding ancestry. As shown in Fig. 1, all the lineages, including individuals representing the same ISFV, formed a distinct clade with a high bootstrap value. The tree showed that the ISFVs can be divided into four main clusters, Culex-hosted ISFVs, including CxFV, *Cx. theileri* flavivirus, YDFV, YNCxFV and QBV; Mansonia-hosted ISFV, only so far containing NAKV; Ochlerotatua-hosted ISFV, comprising with OcFV and HANKV; and Aedes-hosted flaviviruses, including CFAV, AEFV, and KRV. The tree topology indicates that the Shanghai strains belong to the same group of ISFVs, share high sequence identity, and form independent clusters.

### Sequence analysis and phylogenetic characterisation of CxFV genotypes

**Fig. 2:**
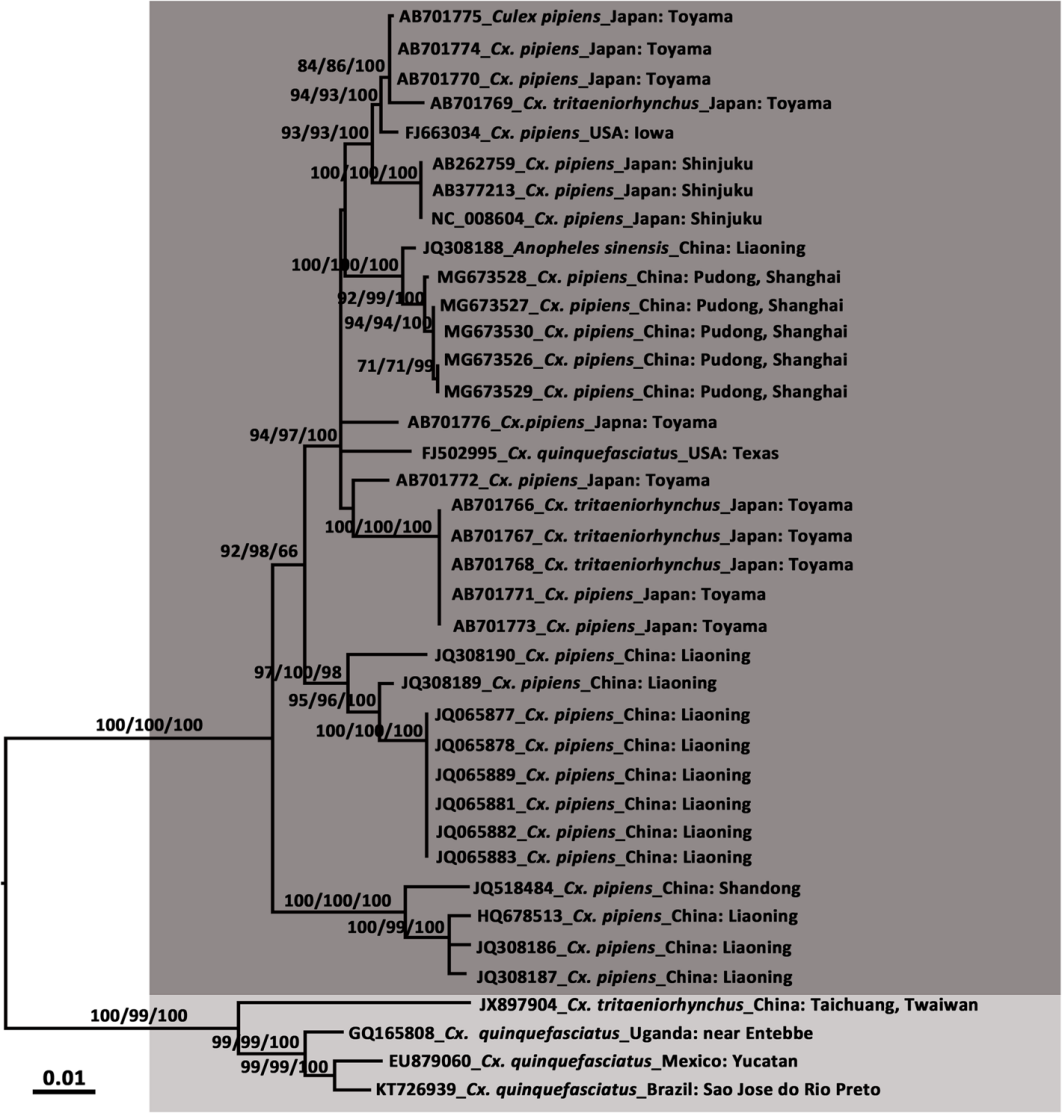
Molecular phylogenetic analysis of the five Culex flavivirus (CxFV) strains based on the nucleotide sequence of the envelope gene. The maximum likelihood tree was constructed by the GTR + I + R model. Bootstrap values (1000 replicates, not shown for less than 60%) of maximum likelihood, neighbour-joining, and Bayesian analyses are shown above the main lineages. The bar indicates 0.01 substitutions per site. Dark grey and light grey indicate CxFV genotype I and genotype II, respectively

The CxFVs were detected in nine pools of *Cx. pipiens*, distributed in Pudong New Area, Chongming District, and Huangpu District. Analysis of their partial NS5 sequence fragments revealed 96.17–97.30% similarity (BLASTn) with the sequences from CxFVs available in GenBank. The CxFV specific primers CxFV-E-F and CxFV-E-R that target a 1443 nt region of the CxFV E gene were used to determine the genotypes of the Shanghai CxFV strains. The amplification of E gene was successful in five of nine CxFV-positive pools. The E sequences of the five pools of mosquitoes showed a high level of identity (99.51–100.00%) with each other, and showed the highest similarity to sequence from the Liaoning DG1064 strain (JQ308188, 99.14–99.51% identity) at the nucleotide level, and lower homology to the Taiwan TW100322 strain (JX897904, 89.35–90.91% identity). The phylogenetic tree presented in Fig. 2 was constructed based on 38 E gene sequences of CxFVs. It was composed of two main monophyletic clades of high confidence. Clade 1 included sequences obtained from Asia and USA, detected primarily in *Cx. pipiens*. Clade 2 comprised CxFVs from Africa, the Caribbean, and Latin America that share the same host, *Cx. quinquefasciatus*. These two clades represented two genotypes. All the CxFV strains from Shanghai that were detected in this paper belong to the former Asia/USA clade. Genetic distance analyses based on E gene sequences showed that, at the nucleotide level, the K2P corrected genetic distances were 0.035 within Clade 1, 0.038 within Clade 2, and 0.113 between the two clades.

Based on the short NS5 sequence fragments, the genetic identity between YNCxFV (KC464457) and CxFVs was 76.98–80.68%, while that between YNCxFV and QBV (NC_012671) was 85.88%. In the NS5 tree (Fig. 1), the YNCxFV sequence presented as a sister group to a group containing several CxFV sequences and the other composited sequences from QBVs.

### Phylogenetic analysis of AEFV sequences

Eleven pools of *Ae. albopictus* tested positive for AEFVs. It is noteworthy that, of these 11 strains, nine were collected from three different streets in Songjiang District in June, and two were collected separately from Qingpu and Huangpu Districts. The results of the pairwise alignment showed that the new strains shared 98.85–100.00% nucleotide identity and were most similar to the AEFV Narita-21 strain (AB488408) from Japan, which shared 90.80–94.64% nucleotide identity with the partial NS5 gene.

### Phylogenetic analysis of QBV sequences

Ten pools tested positive for QBVs. All QBVs described in this paper were detected from pools of *Cx. tritaeniorhynchus* that were collected in Chongming District of Shanghai. The NS5 gene sequences amplified from QBV-positive pools of *Cx. tritaeniorhynchus* share 89.77–91.60% identity with the QBV sequence (FJ644291) from Vietnam.

### Infection rate of ISFVs in mosquitoes from Shanghai

The overall maximum likelihood estimation (MLE) values (and 95% confidence interval, 95% *CI*) of CxFV, QBV, and AEFV, expressed as the number of infected mosquitoes per 1000, for *Cx. pipiens*, *Cx. tritaeniorhynchus*, and *Ae. albopictus* were 1.34 (0.66–2.45), 1.65 (0.87–2.85), and 1.51 (0.77–2.70), respectively. These values indicate the differential spread of ISFVs among the different mosquito species. The infection rate of ISFVs also varied based on district. As shown in Table 2, the MLE-MIR of the AEFVs varies across the districts, with a mean value from 2.42 to 33.93 and an upper limit of 62.14 per 1000 *Ae. albopictus* mosquitoes.

**Table 2:** Maximal likelihood estimation (MLE) and minimum infection rate (MIR) of insect-specific flaviviruses during mosquito activity season of Shanghai in 2016

Detected virus	Survey areas	No. individuals	No. PP	No. pools	Positive pool rate (%)	MLE (95% *CI*)	MIR (95% *CI*)
*Ae. albopictus* mosquito pools							
AeFV	Songjiang District	304	9	40	22.50	33.93 (16.81–62.14)	29.61 (10.55–48.66)
AeFV	Huangpu District	414	1	58	1.72	2.42 (0.14–11.70)	2.42 (0.00–7.14)
AeFV	Qingpu District	119	1	14	7.14	8.42 (0.49–40.88)	8.40 (0.00–24.80)
*Cx. pipiens* mosquito pools							
CxFV	Pudong New Area	232	6	29	20.69	23.05 (8.72–50.60)	21.55 (2.87–40.24)
CxFV	Chongming District	165	1	30	3.33	6.01 (0.35–28.74)	6.06 (0.00–17.90)
CxFV	Huangpu District	109	2	36	5.56	19.39 (3.43–63.88)	18.35 (0.00–43.54)
*Cx. tritaeniorhynchus* mosquito pools							
QBV	Chongming District	2955	10	131	7.63	3.51 (1.81–6.23)	3.38 (1.29–5.48)

*AEFV* Aedes flavivirus, *CxFV* Culex flavivirus, *QBV* Quang Binh virus, *PP* Positive pool, *CI* Confidence interval

## Discussion

The most common fragment of flavivirus genome available in the GenBank database is the widely used NS5 gene. Previous results have shown that the NS5 dataset and topologies are not significantly different from those inferred by the open reading frame; though the bootstrap supporting values at some nodes were lower [21, 28]. The phylogenetic tree of the ISFVs (Fig. 1) suggests divergence of four groups, reflecting sequences isolated from *Aedes*, *Culex*, *Mansonia*, and *Ochlerotatua* mosquitoes. The tree agrees with the hypothesis that each insect flavivirus is maintained in a host genus-associated manner; though the transmission patterns of a few members of this group, like CFAV [8] and CxFV [50], show taxonomic diversity, possibly resulting from frequent host-switching [4].

As evidenced from sequence identity and phylogenetic analysis, one AEFV strains in *Ae. albopictus*, and two CxFV strains in *Cx. pipiens*, captured in Huangpu District; nine AEFV strains in *Ae. albopictus*, captured in Songjiang District; ten QBV strains in *Cx. tritaeniorhynus*, and one CxFV strain in *Cx. pipiens*, captured in Chongming District; one AEFV strains in *Ae. albopictus*, captured in Qingpu District; and six CxFV strains in *Cx. pipiens*, were captured in Pudong New Area of Shanghai. No ISFV-positive mosquitoes were identified in Jiading District. All Shanghai strains of AEFVs, CxFVs, and QBVs were separately collected from mosquitoes belonging to the species *Ae. albopictus*, *Cx. pipiens*, and *Cx. tritaeniorhynchus*, indicating that ISFVs in Shanghai are probably host-related. Reports of ISFVs in China were organised and listed in Additional file 2. In this study, CxFV and QBV, previously detected in other regions of China [13–16, 26, 27], are also found in Shanghai. However, AEFV has not been found in China before, but this study is the first to report AEFV in China. At least, three insect flaviviruses including ISFVs, AEFV, CxFV, and QBV are detected in Shanghai, showing that they are co-circulating in this area. The presence of ISFVs in this area could be explain by the fact that Shanghai is an international and cosmopolite metropolis, where migratory vectors through frequent international trades and human migration could easily introduce insect flaviviruses.

The monthly MLE value (95% *CI*) of AEFV reached 40.18 (20.00–73.57) per 1000 mosquitoes, corresponding to approximately one in three of the homogenates tested. Other researches have also reported high infection rates of mosquitoes by ISFVs in the field [51, 52]. We continued to randomly test collected *Ae. albopictus* for AEFVs from July to October, but did not detect any further AEFV-positive pools in this district. This finding might indicate that the activity of these viruses in the local *Ae. albopictus* population is seasonal, an activity pattern that would support the hypothesis that the ability of ISFVs to transmit may vary seasonally, as proposed by Kim et al. [10]. Two other AEFV-positive pools were collected from Qingpu and Huangpu districts in August and September, respectively. The results of a pairwise alignment showed that these new strains were genetically closely related to the AEFV Narita-21 strain (AB488408) from Japan. This observation indicates that AEFV may have been introduced in Shanghai by tire-travelling tiger mosquitoes.

Culex flaviviruses are widely distributed in the nature and can infect various mosquito species [1, 10–16]. In China, CxFVs have been isolated and/or detected in mosquitoes from Shandong, Liaoning, Gansu, Henan, and Shanxi provinces [15, 16] since they were first reported in 2012 [13]. Unfortunately, the genomic information of the CxFVs isolated from Henan, Shandong, Shanxi, and Gansu provinces is not available in public databases for further phylogenetic analysis. All the CxFVs described here were isolated from *Cx. pipiens*; though CxFVs have occasionally also been isolated from other *Culex* species [10–12, 53], and have been detected in *An. sinensis* in Liaoning Province, China [26]. CxFVs can be divided into two genotypes, the Asia/USA genotype and the Africa/Caribbean/Latin American genotype, according to their E genes [50]. The E genes of the CxFVs in five of the nine CxFV positive pools collected in this study were successfully amplified. Phylogenetic analyses indicated that the CxFVs of Shanghai strains are more closely related to the subtropical lineage than to the tropical one. Based on cytology experiments, the Asia/USA genotype has been reported to cause the cell-fusion type of CPE in C6/36 cells [9, 13, 54]. The Africa/Caribbean/Latin American genotype, in contrast, induces relatively mild CPE, reduced cell density, and modification of cell shape [12], or no observed CPE [1], except for the isolate from Taiwan Province, China, which possesses a glycine residue at position 117 of its E protein. The presence of the glycine residue is a unique character shared by the Asia/USA genotype, which may be associated with the induction of CPE [11]. In this study, all the CxFVs of Shanghai strains had a unique glycine residue at position 117 of their E proteins, which is consistent with the Asia/US genotype.

The Quang Binh virus has been found in southwest Asia in Quang Binh, Vietnam [17] and in Yunnan Province in China [27]. The QBVs detected in this study were solely isolated in *Cx. tritaeniorhynchus* from the Chongming District. Chongming Island has important commercial harbours and a nature reserve in the Dongtan wetland that hosts numerous migratory birds. Migratory vectors through international trade, or wind-blown infected mosquitoes might introduce QBV, as other ISFVs [52].

It is unfortunate that virus isolation could not be done because the mosquitoes were preserved in 75% alcohol. The potential virus in the supernatant of mosquito homogenate cannot be cultivated in mosquito cell lines for further analyses. Thus, the actual infection rate of ISFVs in Shanghai may be underestimated in this study. This study might leverage deep genomic investigations and continuous ISFV mosquito surveillance for a better understanding of the ISFVs circulating in mosquitoes found in Shanghai. In the further, studies might be carried out to investigate any co-effect of ISFV and JEV, or DENV in field caught mosquitoes, as well as the impact on other arboviruses of public health importance such as JEV, DENV, and Zika virus.

## Conclusions

This molecular survey has determined the presence, geographic distribution, genetic variation and infection rate of ISFVs in Shanghai, China. The results showed that, AEFV, CxFV, and QBV are co-circulating in Shanghai, with overall MLE values of 1.51, 1.34, and 1.65 per 1000 of *Ae. albopictus*, *Cx. pipiens*, and *Cx. tritaeniorhynchus*, respectively. Phylogenetic analysis of E gene sequences with those of reference strains has revealed that the Shanghai CxFVs belong to the Asia/USA genotype. Very importantly, this is the first report of AEFV in China.

## Additional files

Additional file 1: Multilingual abstracts in the five official working languages of the United Nations. (PDF 271 kb)

Additional file 2: Summary of the ISFVs documented in mainland China. (PDF 981 kb)

## Abbreviations

AEFV: Aedes flavivirus
bp: Base pair
CFAV: Cell fusing agent virus
*CI*: Confidence interval
CPE: Cytopathic effect
CxFV: Culex flavivirus
DENV: Dengue virus
E gene: Envelope gene
HANKV: Hanko virus
ISFV: Insect-specific flavivirus
JEV: Japanese encephalitis virus
K2P: Kimura’s 2-parameter
KRV: Kamiti River virus
MIR: Minimum infection rate
ML tree: Maximum likelihood tree
MLE: Maximal likelihood estimation
NAKV: Nakiwogo virus
NS5: Nonstructural 5 gene
OcFV: Ochlerotatua flavivirus
QBV: Quang Binh virus
WNV: West Nile virus
YDFV: Yamadai flavivirus
YNCxFV: Yunnan Culex flavivirus
