# Supplementary material for: Co-circulation of Aedes flavivirus, Culex flavivirus, and Quang Binh virus in Shanghai, China
Source: Infect Dis Poverty. 2018 Jul 16;7:75. doi: 10.1186/s40249-018-0457-9 (PMC6052644; doi:10.1186/s40249-018-0457-9)
Supplement: Supplementary file 1 — Multilingual abstracts in the five official working languages of the United Nations. (PDF 271 kb) [file 40249_2018_457_MOESM2_ESM.pdf]

Additional Table. Summary of the insect-specific flaviviruses documented in mainland China

Detected virus	Collect date	Collect location	Host	Strain	GenBank ID			Reference
					ORF	E	NS5	
CxFV	Aug-2006	Dongming, Shandong Province	*Culex pipiens*	SDDM06-11	JQ518484			Wang *et al*., 2012 [1]
YNCxFV	Jul-2009	Lushui, Yunnan Province	*Cx. tritaeniorhynchus*	LSFlaviV-A20-09	KC464457			
YNCxFV	Jun-2009	Nanjian, Yunnan Province	*Cx. tritaeniorhynchus*	YNCxFV-NJ6-09			KC510663	
YNCxFV	Jun-2009	Nanjian, Yunnan Province	*Cx. tritaeniorhynchus*	YNCxFV-NJ5-09			KC510664	
YNCxFV	Jun-2009	Zhenyuan, Yunnan Province	*Anopheles sinensis*	YNCxFV-A125-09			KC510665	
YNCxFV	Jun-2009	Heqing, Yunnan Province	*Cx. tritaeniorhynchus*	YNCxFV-A118-09			KC510666	
YNCxFV	Aug-2009	Malipo, Yunnan Province	*Cx. tritaeniorhynchus*	YNCxFV-A55-09			KC510668	Zuo *et al*., 2014 [2]
YNCxFV	Jul-2009	Mengla, Yunnan Province	*Cx. tritaeniorhynchus*	YNCxFV-A50-09			KC510669	
YNCxFV	Jun-2009	Lushui, Yunnan Province	*Cx. tritaeniorhynchus*	YNCxFV-A11-09			KC510670	
YNCxFV	Jul-2009	Lushui, Yunnan Province	*Cx. tritaeniorhynchus*	YNCxFV-A12-09			KC510671	
YNCxFV	Jul-2009	Lushui, Yunnan Province	*Cx. tritaeniorhynchus*	YNCxFV-A26--09			KC510672	
YNCxFV	Jun-2009	Ninger, Yunnan Province	*Cx. tritaeniorhynchus*	YNCxFV-A39-09			KC510673	
CxFV	Jul-2004	Tanghe, Henan Province	*Cx.* spp.	HNTH04-02				
CxFV	Jul-2004	Tanghe, Henan Province	*Cx.* spp.	HNTH04-03				
CxFV	Jul-2004	Tanghe, Henan Province	*Cx.* spp.	HNTH04-12				
CxFV	Jul-2004	Tanghe, Henan Province	*Cx.* spp.	HNTH04-17				
CxFV	Jul-2004	Tanghe, Henan Province	*Cx.* spp.	HNTH04-19				
CxFV	Jul-2004	Tanghe, Henan Province	*Cx.* spp.	HNTH04-20				Liang *et al*., 2015 [3]
CxFV	Jul-2004	Tanghe, Henan Province	*Cx.* spp.	HNTH04-26				
CxFV	Jul-2004	Tanghe, Henan Province	*Cx.* spp.	HNTH04-28				
CxFV	Jul-2004	Tanghe, Henan Province	*Cx.* spp.	HNTH04-36				
CxFV	Jul-2004	Tanghe, Henan Province	*Cx.* spp.	HNTH04-37				
CxFV	Jul-2004	Tanghe, Henan Province	*Cx.* spp.	HNTH04-41				

CxFV	Jul-2004	Tanghe, Henan Province	*Cx.* spp.	HNTH04-42	
CxFV	Aug-2009	Jiaozhou, Shandong Province	*Cx. pipiens*	SDJZ09-1	
CxFV	Aug-2009	Longkou, Shandong Province	*Anopheles sinensis*	SDLK09-24	
CxFV	Aug-2012	Rizhao, Shandong Province	*Cx. pipiens*	SDRZ12-40	
CxFV	Aug-2012	Rizhao, Shandong Province	*Cx. pipiens*	SDRZ12-44	
CxFV	Aug-2012	Rizhao, Shandong Province	*Cx. pipiens*	SDRZ12-50	
CxFV	Aug-2012	Rizhao, Shandong Province	*Cx. pipiens*	SDRZ12-66	
CxFV	Aug-2012	Junan, Shandong Province	*Cx.. tritaeniorhynchus*	SDJN12-05	
CxFV	Aug-2012	Junan, Shandong Province	*Cx. pipiens*	SDJN12-19	
CxFV	Aug-2012	Junan, Shandong Province	*Cx.. tritaeniorhynchus*	SDJN12-10	Liang *et al*., 2015 [3]
CxFV	Aug-2012	Weishan, Shandong Province	*Cx.. tritaeniorhynchus*	SDJN12-04	
CxFV	Jul-2012	Yanan, Shanxi Province	*Cx. pipiens*	SXYA12-27-2	
CxFV	Jul-2012	Yanan, Shanxi Province	*Cx. pipiens*	SXYA12-27-4	
CxFV	Jul-2012	Yanan, Shanxi Province	*An. sinensis*	SXYA12-27-8	
CxFV	Jul-2012	Yanan, Shanxi Province	*Cx. pipiens*	SXYA12-28-4	
CxFV	Jul-2012	Yanan, Shanxi Province	*Cx. pipiens*	SXYA12-28-6	
CxFV	Jul-2012	Yanan, Shanxi Province	*Cx. pipiens*	SXYA12-28-8	
CxFV	Jul-2012	Yanan, Shanxi Province	*Cx. pipiens*	SXYA12-28-10	
CxFV	Aug-2011	Gansu Province	*Cx. pipiens*	GS11-21	
CxFV	Aug-2011	Gansu Province	*Cx. pipiens*	GS11-38	
CxFV	Aug-2011	Gansu Province	*Cx. pipiens*	GS11-41	
CxFV	Aug-2011	Gansu Province	*Cx. pipiens*	GS11-169	
CxFV	Aug-2011	Gansu Province	*Cx. pipiens*	GS11-170	Zha *et al*., 2012 [4]
CxFV	Aug-2011	Gansu Province	*Cx. pipiens*	GS11-171	
CxFV	Aug-2011	Gansu Province	*Cx. pipiens*	GS11-172	
CxFV	Aug-2011	Gansu Province	*Cx. pipiens*	GS11-173	

CxFV	Aug-2011	Gansu Province	*Cx. pipiens*	GS11-174				
CxFV	Aug-2011	Gansu Province	*Cx. pipiens*	GS11-175				
CxFV	Aug-2011	Gansu Province	*Cx. pipiens*	GS11-176				
CxFV	Aug-2011	Gansu Province	*Cx. pipiens*	GS11-177				
CxFV	Aug-2011	Gansu Province	*Cx. pipiens*	GS11-178				
CxFV	Aug-2011	Gansu Province	*Cx. pipiens*	GS11-179				
CxFV	Aug-2011	Gansu Province	*Cx. pipiens*	GS11-180				Zha *et al*., 2012 [4]
CxFV	Aug-2011	Gansu Province	*Cx. pipiens*	GS11-181				
CxFV	Aug-2011	Gansu Province	*Cx. pipiens*	GS11-182				
CxFV	Aug-2011	Gansu Province	*Cx. pipiens*	GS11-183				
CxFV	Aug-2011	Gansu Province	*Cx. pipiens*	GS11-184				
CxFV	Aug-2011	Gansu Province	*Cx. pipiens*	GS11-185				
CxFV	Aug-2011	Gansu Province	*Cx. pipiens*	GS11-186				
CxFV	Sep-2011	Dandong, Liaoning Province	*Cx. pipiens*	DG1		JQ065883	JQ409188	
CxFV	Sep-2011	Dandong, Liaoning Province	*Cx. pipiens*	DG2		JQ065882	JQ409186	
CxFV	Sep-2011	Dandong, Liaoning Province	*Cx. pipiens*	DG3		JQ065881	JQ409187	An *et al*., 2012 [5]
CxFV	Sep-2011	Dandong, Liaoning Province	*Cx. pipiens*	DG5		JQ065879	JQ409191	
CxFV	Sep-2011	Dandong, Liaoning Province	*Cx. pipiens*	DG6		JQ065877	JQ409189	
CxFV	Sep-2011	Dandong, Liaoning Province	*Cx. pipiens*	DG7		JQ065878	JQ409190	
QBV	Jul-2012	Menghai, Yunnan Province	*Cx.. tritaeniorhynchus*	BNDL1205				Feng *et al*., 2014 [6]
QBV	Jul-2012	Menghai, Yunnan Province	*Cx.. tritaeniorhynchus*	BNDL1227				

YNCxFV, Yunnan Culex flavivirus; CxFV, Culex flavivirus; QBV, Quang Binh virus; ORF: open reading frame; E: envelope gene; NS5: non-structural 5 gene.

References

1. Wang H, Wang H, Fu S, Liu G, Liu H, Gao X-Y, et al. Isolation and identification of a distinct strain of Culex flavivirus from mosquitoes collected in Mainland China. Virol J 2012;9:73-73.
2. Zuo S, Zhao Q, Guo X, Zhou H, Cao W, Zhang J-S. Detection of Quang Binh virus from mosquitoes in China. Virus Res 2014;180:31-38.

3. Liang W, He X, Liu G, Zhang S, Fu S, Wang M, *et al.* Distribution and phylogenetic analysis of Culex flavivirus in mosquitoes in China. Arch Virol 2015;160:2259-2268.
4. Zha B, Yu D-S, Fu S-H, Li G-T, Lv Z, Jiang J-X, *et al.* [Investigation of mosquitoes and arboviruses in Hexi Corridor of Gansu province, China in 2011]. Chin J Vector Biol Control 2012;23:424-427. (In Chinese)
5. An S-Y, Liu J-S, Ren Y, Wang Z-S, Han Y, Ding J, *et al.* [Isolation fo the Culex flavivirus from mosquitoes in Liaoning Province, China]. Chin J Virol 2012;28:511-516. (In Chinese)
6. Feng Y, H.-B. L, J. Z, Zhang Y-Z, Yang W-H, Fan W-H, *et al.* [First isolation of Quang Binh-like virus from mosquitoes in China]. Chin J Virol 2014:57-61. (In Chinese)
