# Supplementary material for: Co-circulation of Aedes flavivirus, Culex flavivirus, and Quang Binh virus in Shanghai, China
Source: Infect Dis Poverty. 2018 Jul 16;7:75. doi: 10.1186/s40249-018-0457-9 (PMC6052644; doi:10.1186/s40249-018-0457-9)
Supplement: Supplementary file 2 — Summary of the ISFVs documented in mainland China. (PDF 981 kb) [file 40249_2018_457_MOESM1_ESM.pdf]

Translation of the abstract into the five official working languages of the United Nations

تتوزع الفيروسة "الزاعجة" المصفرة وفيروسة "البعوضة" المصفرة وفيروس "كوانغ بنه" بشكل مشترك في شنغهاي، الصين

"يوان فانغ" و"يى تشانغ" و"تشنغ بن تشو" و"ون تشى شي" و"شانغ شيا" و"يوان يوان" و"جيا-تونغ وو" و"تشين ليو" و"غوانغ-يي لين"

الملخص
الخلفية: مع انتشار السفر والتجارة العالمية ، لا شك في أن انتشار الأربوفيروسات "الفيروسات المنقولة بالمفصليات" يبعث على القلق. سابقاً : مؤسسة محمد عليان , تسجلت لدى وزارة الصناعة والتجارة في تاريخ 2013/09/03. يمكن للكشف عن مسببات الأمراض في البعوض الذي يتم اصطياده في الميدان أن يقدم التحذير المبكر من انتقال العدوى. تم الكشف لأول مرة عن الفيروس المصفر الخاص بالحشرات (SFVI) في عام 1991 وتم تسجيله في جميع أنحاء العالم في السنوات العشر الأخيرة. على الرغم من أن العدوى بـ"الفيروسات المصفرة الخاصة بالحشرات" تقتصر على الحشرات، إلا أن زيادة معدل الإصابة بالفيروسات المنقولة بواسطة البعوض قد تكون قادرة على إحداث تأثيرات اعتلال خلوي في خلايا الفقاريات أثناء العدوى المشتركة مع مسببات الأمراض البشرية الأخرى. ومع ذلك ، لا يُعرف الكثير عما إذا كانت "الفيروسات المصفرة الخاصة بالحشرات" مستمرة في معظم مناطق الصين.
المناهج: خلال موسم نشاط البعوض في عام 2016، تم تنفيذ برنامج مراقبة لاكتشاف "الفيروسات المصفرة الخاصة بالحشرات" في البعوض في شنغهاي الحضرية، الصين. تم اختبار وجود "الفيروسات المصفرة الخاصة بالحشرات" بشكل عشوائي في أنواع مختلفة من البعوض باستخدام مقايسات تفاعل البوليميراز المتسلسل القائمة على PCR-RT والمتداخلة جزئيًا، متبوعة بتسلسل منتجات تفاعل البوليميراز المتسلسل. وتمت مقارنة التسلسلات من العينات المجمعة الإيجابية مع تلك المودعة في بنك "جِن-بنك". بعد ذلك، تم استخدام سلاسل من فيروسات الحشرات التمثيلية الحشرية لمزيد من التحليلات التطورية السارية والجزيئية.
النتائج: أظهرت تحقيقاتنا: (1) وجود الفيروسة "الزاعجة" المصفرة (AEFV) في 161/11 عينات مجمعة (تسع بركس في منطقة سونغ جيانغ، وبركة واحدة في مقاطعة هوانغبو، وبركة واحدة في منطقة كوينغبو) للزاعجة المنقطة بالأبيض. (2) وجود فيروس كوانغ بنه (QBV) في 195/10 عينات مجمعة (كلها في منطقة تشونغمينغ) للباعضة الشريطيةُ الأنف. (3) وجود فيروسة "البعوضة" المصفرة (CxFV) في عينات مجمعة 228/9 (ستة حمامات في منطقة بودونغ الجديدة، ومسبحين في منطقة هوانغبو، وبركة واحدة في منطقة تشونغمينغ) للبعوضة المنزلية الشمالية. وعلاوة على ذلك، أشارت التحليلات الجينية للتسلسل الجيني لبروتينات المغلفات إلى أن سلالات فيروسة "البعوضة" المصفرة بشنغهاي تنتمي إلى النمط الوراثي لآسيا / الولايات المتحدة الأمريكية. كانت القيم الإجمالية لتقدير احتمالية الحد الأقصى (وفاصل الثقة 95%) لـ CxFV و QBV و AEFV في البعوض التي تم جمعها في شنغهاي في عام 2016 هي 1.34 (0.66–2.45) و 1.65 (0.87–2.85) و 1.51 (0.77–2.70) لكل 1000، على التوالي.
الاستنتاجات: تكشف هذه الدراسة عن وجود الفيروسات المصفرة الخاصة بالحشرات والتوزيع الجغرافي لها، وتحدد التباين الوراثي ومعدل الإصابة بالفيروسات المصفرة الخاصة بالحشرات في شنغهاي، الصين. على الأقل، ثلاثة أنواع من فيروسات الفلاف الحشرية ، بما في ذلك الفيروسات المصفرة الخاصة بالحشرات والفيروسة "الزاعجة" المصفرة AEFV وفيروسة "البعوضة" المصفرة CxFV وفيروس كوانغ بنه QBV ، يتم توزيعها في هذه المنطقة. على حد علمنا ، هذا هو أول تقرير عن الفيروسة "الزاعجة" المصفرة AEFV في الصين.

Translated from English version into Arabic by Mohamed Hamdi, proofread by Mohamed Abo-Sahal, through

TRANSLATORS
WITHOUT BORDERS

伊蚊黄病毒、库蚊黄病毒、广平病毒在上海共同流行

方圆、张仪、周正斌、施文琦、夏尚、李元元、吴嘉彤、刘琴、林光一

摘要
引言：随着国际间商贸往来、旅游、及参与其他国际交流活动的人员日益频繁，媒传病毒的传播和扩散速度备受关注。对蚊媒传染病进行有效监控可在早期对这类传染病的流行提供预警。继昆虫特有黄病毒首次在 1991 年发现，近十年在世界各地均有昆虫特有黄病毒的陆续报道。虽然昆虫特有黄病毒只在蚊虫体内传播，但当其与其他人类病原体共同感染时，昆虫特有黄病毒可能会引起脊椎动物细胞病变。然而目前昆虫特有黄病毒在我国许多地方的分布调查仍存在空白。
方法：本项目于 2016 年蚊媒活动的高峰期对上海地区蚊媒进行昆虫特有黄病毒监测。基于 RT-PCR 和半巢氏 PCR 对不同蚊种进行了昆虫特有黄病毒抽样检测，PCR 阳性产物经测序与 GenBank 上的序列进行比对和系统进化分析。
结果：调查结果显示，在抽样检测的 161 份白纹伊蚊中发现 11 份样本为伊蚊黄病毒阳性（松江区 9 份、黄浦区 1 份，青浦区 1 份伊蚊黄病毒阳性样本）；在抽样检测的 195 份三带喙库蚊中发现 10 份样本为广平病毒阳性，均分布在崇明区；在抽样检测的 228 份淡色库蚊中发现 9 份样本

为库蚊黄病毒阳性（浦东新区 6 份、黄浦区 2 份，崇明区 1 份库蚊黄病毒阳性样本）。进而通过 E 蛋白编码序列比对和系统进化分析，鉴定本研究在上海所检测到的库蚊黄病毒属亚洲/北美基因型。使用最大似然法所估算的库蚊黄病毒、广平病毒和伊蚊黄病毒在相应媒介中的感染率分别为 1.34 (0.66–2.45)、1.65 (0.87–2.85)、1.51 (0.77–2.70) 每千只蚊虫。
结论：本研究对上海市昆虫特有黄病毒进行了种类、分布等调查，并对所属基因型和感染率等作了进一步的分析和评估。确定了目前在上海地区至少存在伊蚊黄病毒、库蚊黄病毒和广平病毒三种昆虫特有黄病毒的共同流行。其中伊蚊黄病毒为我国的新纪录。

Translated from English version into Chinese by Yuan Fang

Cocirculation des flavivirus transmis par les moustiques des genres *Aedes* et *Culex* et du virus Quang Binh à Shanghai (Chine)

Yuan Fang, Yi Zhang, Zheng-Bin Zhou, Wen-Qi Shi, Shang Xia, Yuan-Yuan Li, Jia-Tong Wu, Qin Liu, Guang-Yi Lin

Résumé
Contexte: Le caractère alarmant de la propagation des arbovirus, facilitée par l'expansion du commerce international et l'augmentation des déplacements à l'échelle mondiale, ne fait aucun doute. La détection des agents pathogènes chez les moustiques prélevés sur le terrain permet d'alerter le plus tôt possible sur un risque de transmission. Les flavivirus spécifiques d'insectes (FSI) ont été détectés pour la première fois en 1991 et sont documentés, dans le monde entier, depuis une dizaine d'années. Bien que l'infection à FSI soit, a priori, limitée aux insectes, il est possible qu'une augmentation du taux d'infections à flavivirus transmises par les moustiques induise des effets cytopathiques sur les cellules des vertébrés lors d'une co-infection avec d'autres pathogènes humains. Les données sur la persistance éventuelle des FSI dans la plupart des régions de Chine sont cependant limitées.
Méthodes: Pendant la saison d'activité des moustiques en 2016, un programme de surveillance a été mis en œuvre pour détecter la présence de FSI chez les moustiques dans l'aire urbaine de Shanghai, en Chine. La présence de FSI a été recherchée, de manière aléatoire, chez différentes espèces de moustiques au moyen de dosages par RT-PCR et PCR semi-nichée, dont les produits ont ensuite été séquencés. Les séquences des pools d'échantillons positifs ont été comparées à celles enregistrées dans la base de données GenBank. Les séquences de flavivirus d'insectes représentatifs ont ensuite été utilisées pour des analyses d'évolution moléculaire et phylogénétique.
Résultats: Nos investigations ont mis en évidence: (1) la présence de flavivirus transmis par les moustiques du genre *Aedes* (AEFV) dans 11 des 161 échantillons des pools (neuf pools dans le district de Songjiang, un dans le district de Huangpu et un dans le district de Qingpu) d'*Aedes albopictus*, (2) la présence du virus Quang Binh (QBV) dans 10 des 195 échantillons des pools (tous dans le district de Chongming) de *Culex tritaeniorhynchus* et (3) la présence de flavivirus transmis par les moustiques du genre *Culex* (CxFV) dans 9 des 228 échantillons des pools (six pools dans la Zone Nouvelle de Pudong, deux dans le district de Huangpu et un dans le district de Chongming) de *Cx. pipiens*. Par ailleurs, les analyses phylogénétiques des séquences géniques des protéines d'enveloppe ont révélé que les souches de CxFV retrouvées à Shanghai étaient de génotype asiatique/étatsunien. Les valeurs globales d'estimation de la probabilité maximale (et l'intervalle de confiance à 95 %) de présence de CxFV, de QBV et d'AEFV chez les moustiques prélevés à Shanghai en 2016 étaient respectivement de 1,34 ‰ (0,66–2,45), 1,65 ‰ (0,87–2,85) et 1,51 ‰ (0,77–2,70).
Conclusions: Cette étude révèle la présence et la distribution géographique des FSI et détermine la variation génétique et le taux d'infection à FSI à Shanghai, en Chine. Au moins trois flavivirus d'insectes (dont les FSI AEFV, CxFV et QBV) cocirculent dans cette région. À notre connaissance, il s'agit du premier signalement d'AEVF en Chine.

Translated from English version into French by CcilJ, proofread by Suzanne Assenat, through

TRANSLATORS
WITHOUT BORDERS

Параллельная циркуляция флавивирусов Aedes, Culex и Quang Binh в Шанхае, Китай

Юань Фан, И Чжан, Чжэн-Бинь Чжоу, Вэнь-Ци Ши, Шан Ся, Юань-Юань Ли, Цзя-Тун У, Цинь

Лю, Гуан-И Линь

Аннотация
Краткое описание: С увеличением числа международных поездок и расширением торговли распространение арбовирусов бесспорно вызывает тревогу. Выявление патогенов у комаров в полевых условиях может послужить наиболее ранним предупреждением о передаче инфекции. Специфический для насекомых флавивирус (ISFV) был впервые обнаружен в 1991 году и за последние десять лет зарегистрирован по всему миру. Несмотря на то, что заражение вирусами ISFV, по всей видимости, ограничивается только насекомыми, рост уровня заболеваемости переносимых комарами флавивирусами может вызывать цитопатический эффект в клетках позвоночника при одновременном инфицировании другими патогенами человека. Тем не менее, о том, что вирусы ISFV все еще встречаются в большинстве регионов Китая, известно мало. **Методы:** Во время сезона активности комаров в 2016 году была проведена программа обследования по выявлению вирусов ISFV у комаров в городских районах Шанхая, Китай. Различные виды комаров были случайным образом проверены на наличие вирусов ISFV при помощи методов ОТ-ПЦР и полугнездовой ПЦР с последующим секвенированием продуктов ПЦР. Последовательности из пулированных проб с положительными результатами сравнили с образцами, хранящимися в базе данных GenBank. В дальнейшем, последовательности флавивирусов репрезентативных насекомых использовались для филогенетических и молекулярно-эволюционных анализов. **Результаты:** Наши исследования показали: (1) наличие флавивируса Aedes (AEFV) в 11 пулированных пробах (девять пулов в районе Сунцзян, один пул в районе Хуанпу и один пул в районе Цинпу) из 161 пробы комаров вида *Aedes albopictus*, (2) наличие вируса Quang Binh (QBV) в 10 из 195 пулированных проб (все в районе Чунмин) комаров вида *Culex tritaeniorhynchus*; и (3) наличие флавивируса Culex (CxFV) в 9 из 228 пулированных проб (шесть пулов в Новом Районе Пудун, два пула в районе Хуанпу и один пул в районе Чунмин) комаров вида *Cx. pipiens*. Кроме того, филогенетические анализы генных последовательностей оболочечных белков указывают на принадлежность штаммов вируса CxFV, выявленного в Шанхае, к генотипу, встречающемуся в Азии и США. Общие значения оценок по максимуму правдоподобия (и интервал при доверительной вероятности 95%) для CxFV, QBV и AEFV у комаров, собранных в Шанхае в 2016 году, составили соответственно 1,34 (0,66–2,45), 1,65 (0,87–2,85) и 1,51 (0,77–2,70) на 1000.
Выводы: Данное исследование выявило присутствие и географическое распределение вирусов ISFV, а также определило генетическую изменчивость и уровень зараженности вирусами ISFV в Шанхае, Китай. По крайней мере, три переносимых насекомыми флавивируса AEFV, CxFV и QBV, относящихся к типу ISFV, параллельно циркулируют в этой области. Насколько нам известно, это первый доклад о флавивирусе AEFV в Китае.

Translated from English version into Russian by Natalia Potashnik, proofread by Tatiana Kary, through

Circulación del flavivirus Aedes, el flavivirus Culex y el virus Quang Binh en Shanghái, China

Yuan Fang, Yi Zhang, Zheng-Bin Zhou, Wen-Qi Shi, Shang Xia, Yuan-Yuan Li, Jia-Tong Wu, Qin Liu, Guang-Yi Lin

Resumen
Contexto: La propagación de los arbovirus es sin duda alarmante con los aumentos del turismo y comercio mundial. La detección del agente patógeno en los mosquitos capturados en terreno puede ser la advertencia más temprana de transmisión. Los flavivirus insecto-específicos (ISFV, por sus siglas en inglés) se detectaron por primera vez en 1991 y se han documentado en todo el mundo durante los últimos diez años. Aunque la infección que causan los ISFV está supuestamente limitada a los insectos, un aumento en el índice de infección con los flavivirus trasmitidos por mosquitos podría inducir efectos citopáticos en células de vertebrados durante la co-infección con otros agentes patógenos humanos. Sin embargo, se sabe poco acerca de si persisten los ISFV en la mayoría de las regiones de China.
Métodos: Durante la temporada de actividad de los mosquitos en 2016, se llevó a cabo un programa de observación para detectar los ISFV en mosquitos en el área metropolitana de Shanghái, China. Se analizó de manera aleatoria la presencia de los ISFV en diferentes especies de mosquitos utilizando

ensayos de RT-PCR y PCR hemianidada, seguidos por la secuenciación de los productos de la PCR. Se compararon las secuencias de muestras conjuntas positivas con las que estaban depositadas en GenBank. A partir de ahí, se utilizaron las secuencias de flavivirus de insectos representativos para más análisis filogenéticos y de evolución molecular.
Resultados: Nuestras investigaciones demostraron: (1) la presencia del flavivirus Aedes (AEFV) en 11 de 161 muestras conjuntas (nueve conjuntos en el distrito de Songjiang, un conjunto en el distrito de Haungpu y un conjunto en el distrito de Qingpu) de *Aedes albopictus*, (2) la presencia del virus Quang Binh (QBV) en 10 de 195 muestras conjuntas (todas en el distrito de Chongming) de *Culex tritaeniorhynchus*y (3) la presencia del flavivirus Culex (CxFV) en 9 de 228 muestras conjuntas (seis conjuntos en Pudong New Area, dos conjuntos en el distrito de Huangpu y un conjunto en el distrito de Chongming) de *Cx. pipiens*. Además, los análisis filogenéticos de las secuencias genéticas de las proteínas de envolturas mostraron que las cepas del CxFV de Shanghái pertenecían al genotipo de Asia/EE.UU. Los valores generales máximos de probabilidad estimada (y 95 % del intervalo de confianza) para CxFV, QBV y AEFV en los mosquitos reunidos en Shanghái en 2016 fueron de 1,34 (0,66-2,45), 1,65 (0,87-2,85) y 1,51 (0,77-2,70) por cada 1000, respectivamente.
Conclusiones: Este estudio revela la presencia y la distribución geográfica de los ISFV y determina la variación genética y el índice de infección de los ISFV en Shanghái, China. Al menos tres flavivirus de insectos, incluidos los ISFV, AEFV, CxFV y QBV, circulan en esta área. Según nuestro conocimiento, este es el primer informe de AEFV en China.

Translated from English version into Spanish by Maximiliano Juncos, proofread by Mayra León, through

TRANSLATORS
WITHOUT BORDERS
